# Taeumjowi-tang, a Traditional Korean Sasang Remedy, Improves Obesity-Atopic Dermatitis Comorbidity by Regulating Hypoxia-Inducible Factor 1 Alpha

**DOI:** 10.3389/fphar.2019.01458

**Published:** 2019-12-20

**Authors:** Jinbong Park, Dong-Hyun Youn, JongWook Kang, Kwang Seok Ahn, Hyun Jeong Kwak, Jae-Young Um

**Affiliations:** ^1^ Department of Pharmacology, College of Korean Medicine, Kyung Hee University, Seoul, South Korea; ^2^ Comorbidity Research Institute, Kyung Hee University, Seoul, South Korea; ^3^ Department of Science in Korean Medicine, Graduate School, Kyung Hee University, Seoul, South Korea; ^4^ Department of Life Science, College of Natural Sciences, Kyonggi University, Suwon, South Korea

**Keywords:** comorbidity, atopic dermatitis, obesity, Taeumjowi-tang, hypoxia-inducible factor 1 alpha

## Abstract

Atopic dermatitis (AD) is an inflammatory disease of the skin, resulting from an immune dysfunction, that often occurs as a comorbidity of obesity. This investigation evaluated the capacity of Taeumjowi-tang (TJT), a Korean herbal formulation from the Sasang medical tradition to influence prognostic features of AD and obesity in a mouse model. Here, obesity and AD were induced by a high-fat diet (HFD) and 1-fluoro-2,4-dinitrobenzene (DNFB). Following an 8-week HFD regimen and 4 weeks of DNFB administration, the comorbid (CO) group manifested increased body weight and AD-like lesions, as compared to normal control (NC) mice, while TJT administration diminished these symptoms of obesity and AD. Specifically, TJT treatment reduced epidermal thickness and eosinophil/mast cell infiltration, along with reduction in immunoglobulin E, interleukin (IL)-4, IL-6, and tumor necrosis factor-alpha (TNF-α). It was additionally demonstrated that TJT suppresses HFD/DNFB-associated increase of the inflammation-related nuclear factor-kappa beta (NF-κB) and mitogen activated protein kinase. Moreover, significantly increased levels of hypoxia inducible factor-1 alpha (HIF-1α) protein was observed in CO group versus controls, an increase significantly down-regulated by TJT-treatment. These outcomes suggest that TJT may prove useful in clinical management of obesity-AD comorbidity treatment, an effect that may be due to regulation of HIF-1α expression.

## Introduction

Atopic dermatitis (AD) is an inflammatory skin disease, characterized by continuously relapsing eczematous lesions (Tollefson and Bruckner, 2014). Over 20% of children in industrialized countries are afflicted with AD and global prevalence of the disorder is on the increase at the time of this writing, especially in developing nations (Flohr and Mann, 2014). AD is a known co-morbidity of obesity, which is defined as a condition of ‘over-nutrition’, in which energy intake through diet, exceeds energy expenditure, resulting in storage of surplus energy in adipocytes which accumulates as fat deposits (Park et al., 2013a). Obesity has emerged as a public health challenge, particularly in affluent nations in which sedentary lifestyles combined with overnutrition exacerbate the problem. According to a report by the World Health Organization, over 1.4 billion individuals aged 20 years or older worldwide are overweight (WHO, 2018).

AD and obesity are two totally different diseases clinically. However, the prevalence of the comorbidity of these two diseases is reported to be quite high. According to the National Health and Nutrition Examination Survey of 2005 to 2006, overweight children and adolescents had a higher incidence of AD than that of normal weight children (Visness et al., 2009). Relevant to this, the National Survey of Children’s Health in 2007–2008 reported that the prevalence of overweight and obesity were increased in adolescents with AD compared to adolescents without AD (Silverberg and Simpson, 2014). However, despite the epidemiological relevance of these two diseases, the underlying mechanism is not fully understood. Some studies indeed attempted to search for the link between obesity and AD. While Hooper and Hooper suggest heat shock proteins as the main factor (Hooper and Hooper, 2009), Jeong et al. offer adipokines have a key role (Jeong et al., 2015). On the other hand, Savetsky and colleagues suggest the impaired lymphatic function is the main cause (Savetsky et al., 2015). Although most researchers agree that inflammation is the main mechanism which links the two diseases, the detailed mechanism has not been elucidated clearly.

In Sasang constitutional medicine, a unique field of Traditional Korean Medicine, people are categorized into four types based on their structural and functional variations: Taeyang, Taeum, Soyang, and Soeum (Lee et al., 2009a). Among them, a Taeum type person theoretically show higher vulnerability to obesity-allergy comorbidity than other three types. Taeumjowi-tang (TJT) is an herbal medication introduced by Lee Je-ma in Donguisusebowon. TJT, consists of eight herbs and originally is prescribed to treat stomach-related symptoms in Taeum type persons (Park et al., 2012). However, theoretical possibility of the application of this remedy to obesity and AD leads to clinical use of TJT nowadays. Several studies have reported that TJT showed anti-obese effects in rodents (Park and Cho, 2004; Lee et al., 2009b), and a 12-week trial of 102 participants revealed its potential as a possible safe anti-obesity treatment (Park et al., 2013b). TJT may also be prescribed for AD in Taeum type patients, and Sun et al. have even shown some positive results from a follow-up study (Sun et al., 2004). However, its effect on obesity-AD comorbidity has not been reported to date.

Because we expected TJT to be an efficient treatment for obesity-AD comorbidity, we established a basic level obesity-AD comorbidity mouse model by feeding high fat diet (HFD) while administrating 1-fluoro-2,4-dinitrobenzene (DNFB), an immune sensitizing agent widely used to study AD-like contact dermatitis (Galli and Tsai, 2012). Then, the effect of TJT was confirmed by assessing the histomorphological changes, mast cell infiltration, and also the AD-related markers in the serum and skin. Furthermore, we evaluated the role of hypoxia-inducible factor-1 alpha (HIF-1α) in obesity-AD comorbidity, and then investigated whether it is regulated by TJT treatment.

## Materials and Methods

### Ethics Statement

All experimental protocols involving the use of animals conform to the NIH guidelines (Guide for the Care and Use of Laboratory Animals, 8th edition). Animal Care and Use Committee of the Institutional Review Board of Kyung Hee University (confirmation number: KHUASP(SE)-12-036) has approved all the animal experiments.

### Chemical Reagents

TJT was purchased from I-World Pharm. Co. (Incheon, Republic of Korea). The herbal constituents of TJT are shown in **Table 1**. The antibodies for p38 (sc-7149), NF-κB (sc-372), p-IκB (sc-8404), histone H3 (sc-10809), and glyceraldehyde-3-phosphate dehydrogenase (GAPDH) (sc-32233) were from Santa Cruz Biotechnology (Santa Cruz, CA, USA). Antibodies for p-p38 (#4511), p-ERK (#4695), p-JNK (#9255), and JNK (#9252) were purchased from Cell Signaling Technology (Danvers, MA, USA). Anti-ERK antibody (AP0484) was obtained from Bioworld Technology (St. louis Park, MN, USA), and anti-HIF-1α antibody (610958) was from BD Bioscience (San Jose, CA, USA). Dulbecco’s modified Eagle’s medium (DMEM), RPMI 1640 medium penicillin/streptomycin/glutamine and bovine serum (BS) were from Gibco BRL (Grand Island, NY, USA). Fetal bovine serum (FBS) was purchased from HyClone (Logan, UT, USA). Control siRNA (sc-37007) and siHIF1A (sc-35561) were purchased from Santa Cruz Biotechnology (Santa Cruz, CA, USA), and LPS was from Sigma Aldrich Inc. (St Louis, MO, USA).

**Table 1:** Herbal constituents of TJT (of total 25 mg).

Herbal constituent	Taxonomic name	Dose
Coicis semen	*Coix lacryma-jobi* var. *ma-yuen (Rom.Caill.) Stapf*	3.75 mg
Castaneae semen	*Castanea crenata Siebold & Zucc.*	3.75 mg
Raphani semen	*Raphanus raphanistrum subsp. sativus (L.) Domin*	2.5 mg
Schisandrae fructus	*Schisandra chinensis (Turcz.) Baill.*	1.25 mg
Platycodi radix	*Platycodon grandiflorus (Jacq.) A.DC.*	1.25 mg
Acori graminei rhizoma	*Acorus gramineus Aiton*	1.25 mg
Ephedra herba	*Ephedra sinica Stapf*	1.25 mg
Liriopis tuber	*Liriope muscari (Decne.) L.H.Bailey*	1.25 mg

### Ultra Performance Liquid Chromatography (UPLC) Analysis

Liquid chromatography-mass spectrometry system consisted of a Thermo Scientific Vanquish UHPLC system (ThermoFisher Scientific, CA, USA) with Poroshell 120 EC-C18 (2.1 x 100 mm, 2.7 μm) column (Agilent) and a triple ToF 5600+ mass spectrometer system (Triple ToF MS) (SCIEX, Foster City, CA, USA). Triple TOF MS, equipped with a DuosprayTM ion source, was used to complete the high resolution experiment.

### Animal Experiments

Four-week-old male C57BL/6J mice weighing 17–18 g were purchased from the Dae-Han Experimental Animal Center (Eumsung, Republic of Korea). The mice were maintained for 1 week prior to the experiments in a 12-h light/dark cycle at a humidity of 70% and a constant temperature of 23 ± 2°C. After acclimation, the animals were divided into five groups (n = 7 per group): (a) a normal control (NC) group fed normal chow diet (CJ Feed Co., Ltd., Seoul, Republic of Korea); (B) an AD (DNFB) group fed normal chow diet and applied with DNFB 3 times/week, starting on week 5; (C) and obesity (HFD) group fed with 60% high-fat diet (HFD) (Rodent diet D12492, Research diet, New Brunswick, NJ, USA) for 8 weeks; (D) an obesity-AD comorbidity (CO) group fed a HFD for 8 weeks with a 4-week-DNFB application starting on week 5; and (E) a TJT group which were fed a HFD for 4 weeks to induce obesity, and then fed for 4 additional weeks with HFD plus TJT mixed in diet (125 mg/kg/day) while DNFB was applied starting on week 5, same as the CO group. DNFB sensitization was induced by repeated application of 150 μl of 0.35% DNFB in acetone-olive oil combination (3:1) on the shaved dorsal skin three times a week. The body weight and food intake amount were recorded every other day. At the end of the experiment, the animals were fasted overnight, anesthetized with CO_2_ asphyxiation, and the dorsal skin was removed and divided in half; one half was fixed in 10% formalin and embedded in paraffin for histomorphological assays, and the other half was stored at -80°C for further assays.

### Serum Analysis

Serum was separated by centrifugation at 4,000×*g* for 30 min immediately after blood collection *via* cardiac puncture. Total cholesterol, low density lipoprotein (LDL) cholesterol, triglyceride, alanine aminotransferase (ALT), aspartate aminotransferase (AST), and creatinine levels were assessed using enzymatic colorimetric methods performed by Seoul Medical Science Institute (Seoul Clinical Laboratories, Seoul, Korea).

### Dermatitis Score

Severity of AD-like lesions was evaluated according to the SCORAD (SCORing Atopic Dermatitis) index (Oranje et al, 2007). This scoring was based on the severity of erythema, edema/papulation, oozing/crusts, excoriations, and lichenification each on the scale from 0 to 3 (0, none; 1, mild; 2, moderate; 3, severe). Overall score was determined by summing all individual scores. The SCORAD assessment was performed after group blinding.

### Hematoxylin and Eosin (H&E) Staining, Toluidine Blue Staining, and Immunofluorescence (IF) Assay

The dorsal skin specimens were prepared in 5-μm-thick formalin-fixed, paraffin-embedded tissue sections as previously described (Youn et al., 2017). Five slides per mouse were prepared for the analyses. The sections were deparaffinized in xylene and rehydrated in serial alcohol. After treatment of 150 μl of a 0.1% trypsin working solution (consisting of trypsin 0.4 mL, calcium 0.01 g, and chloride 0.01 g in D.W. 7 mL) for 15 min, the sections were blocked using fetal bovine serum (FBS). For H&E staining, the sections were stained in hematoxylin for 5 min, and then washed with water for 5 min, followed by 30 s of eosin staining. For toluidine blue staining, the sections were stained in toluidine blue for 5 h, and then washed with water. For the IF assay, sections were incubated in 4°C overnight with a 1:50 dilution of the primary antibody and then incubated at room temperature for 30 min with a 1:500 dilution of the Alexa Fluor 633 conjugate (Pierce Thermo Scientific, Rockford, IL, USA). Then, the sections were dehydrated and mounted by routine methods. Five slides for each group were randomly chosen and analyzed by a researcher who was blinded from the experiment. The slides were examined using the Olympus IX71 Research Inverted Phase microscope (Olympus Co., Tokyo, Japan), and the density was measured with the ImageJ 1.47v software (National Institute of Health, Bethesda, MD, USA).

### RNA Extraction and Real-Time RT-PCR

RNA extraction of dorsal skin was performed using the GeneAllR RiboEx total RNA extraction kit (GeneAll Biotechnology, Seoul, South Korea), and Real-time RT-PCR was performed with the Power cDNA synthesis kit (iNtRON Biotechnology, Seongnam, Kyunggi, South Korea), SYBR Green Power Master Mix (Applied Biosystems, Foster City, CA, USA) and the Step One Real-Time PCR System (Applied Biosystems, Foster City, CA, USA) according to the manufacturers’ instructions as previously described (Lim et al., 2016). The primers used in this study are shown in **Table 2**.

**Table 2:** Primer information used in this study.

Gene name	Sequence	Product size	Accession no.
*mTnfa*	Sense: 5′-CTGTGAAGGGAATGGGTGTT-3′ Antisense: 5′-GGTCACTGTCCCAGCATCTT-3′	180 bp	U69613.1
*mIl4*	Sense: 5′-ACAGGAGAAGGGACGCCAT-3′ Antisense: 5′-GAAGCCCTACAGACGAGCTCA-3′	74 bp	NM_021283.2
*mIl6*	Sense: 5′-GAGGATACCACTCCCAACAGACC-3′ Antisense: 5′-AAGTGCATCATCGTTGTTCATACA-3′	117 bp	NM_031168.2
*mGapdh*	Sense: 5′-CCAGGTTGTCTCCTGCGACT-3′ Antisense: 5′-ATACCAGGAAATGAGCTTGACAAAGT3′	80 bp	NM_001289726.1
*mHif1a*	Sense: 5′-AGCTTCTGTTATGAGGCTCACC-3′ Antisense: 5′-TGACTTGATGTTCATCGTCCTC-3′	369 bp	NC_000078.6

### Enzyme-Linked Immunosorbent Assay (ELISA)

The ELISA assay was performed under modifications of methods by Kim et al. (2017). Skin tissue was homogenized in 1.5 mL extraction buffer (containing 10 mM Tris pH 7.4, 150 mM NaCl, 1% Triton X-100) per gram of tissue, centrifuged at 13,000×*g* for 10 min at 4°C, and the supernatant was used for ELISA analysis. Serum IgE was measured with a mouse IgE ELISA kit (Abcam Inc., Cambridge, MA, USA) from total serum samples (n = 7 per group), and TNF-α and IL-6 expressions of the dorsal skin tissues were measured with a mouse TNF-α ELISA kit (Pierce Thermo Scientific, Rockford, IL, USA) and a mouse IL-6 ELISA kit (BD Bioscience, San Jose, CA, USA). Color development was measured by a VERSAmax microplate reader (Molecular Devices, Sunnyvale, CA, USA) at 450 nm.

### Western Blot Analysis

Western blot analyses were performed as described previously (Lee et al., 2016). Briefly, prepared skin tissues were cut into pieces and homogenized with the Bullet Blender homogenization kit (Next Advance Inc., Averill Park, NY, USA). Homogenized tissues were lysed in ice-cold RIPA buﬀer. Nuclear extracts for NF-κB evaluation were prepared using NE-PERTM Nuclear and Cytoplasmic Extraction Reagents (Pierce Thermo Scientific, Rockford, IL, USA). After the protein concentration determination, equal amounts of total protein were resolved by 6–12% SDS polyacrylamide gel electrophoresis and then transferred to a polyvinylidene difluoride (PVDF) membrane. After an overnight incubation with the primary antibodies (NF-κB, p-ERK, ERK, p-JNK, JNK, p-p38, p38, and HIF-1α, the blots were then incubated with proper horseradish peroxidase (HRP)-conjugated secondary antibodies (Jackson Immuno Research, West Grove, PA, USA) for 1 h at RT. The chemiluminescent intensities of the protein signals were quantified with the ImageJ 1.47v software (National Institute of Health, Bethesda, MD, USA).

### Adipocyte Cell Culture, Differentiation, and Conditioned Medium (CM) Preparation

Murine 3T3-L1 mouse embryo ﬁbroblasts (American Type Culture Collection, Rockville, MD, USA) were cultured in 10% BS plus DMEM and differentiated in 10% FBS plus DMEM containing 0.5 mM IBMX, 1 μM dexamethasone and 1 μg/mL insulin (MDI) at 37°C, 5% CO2 as in a previous report (Lim et al., 2016). After 8 day of full differentiation into mature adipocytes, the cells were discarded, and culture media was saved for CM application.

### Establishment of Obesity-AD Comorbidity *In Vitro* Model and siRNA Transfection

Human keratinocytes HaCaT cells were purchased from CLS CellLines Service (Eppelheim, Baden-Württemberg, Germany). HaCaT cells were cultured in RPMI 1640 supplemented with 10% FBS and penicillin/streptomycin in a humidified atmosphere of 37°C, 5% CO_2_ as previously described (Kee et al., 2017) 59.5 μg/mL of LPS was treated for 48 h to HaCaT cells cultured in 50% adipocyte CM in fresh culture medium to mimic an obesity-AD condition. TJT was treated for 24 h, starting at 24 h after CM supplementation. siHIF1A was applied for 24 h prior to TJT treatment following the manufacturer’s instructions.

### Statistical Analysis

Data were expressed as the mean ± standard error of the mean (SEM). Significant differences between the groups were determined with the Student’s t-test and one-way ANOVA followed by post-hoc Tukey’s multiple comparisons tests. All statistical analyses were performed with SPSS statistical analysis software version 11.5 (SPSS Inc., Chicago, IL, USA). A probability value of *P* < 0.05 was considered as statistical significant.

## Results

### Chromatographic Analysis of TJT

**Figure 1:**
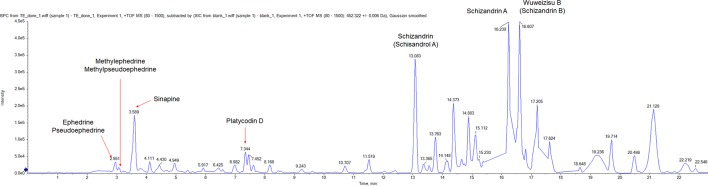
The UPLC analysis of TJT. The representative chromatogram of TJT and identification of its components. Ephedrine, pseudoephedrine, methylephedrine, methylpseudoephedrine, sinapine, platycodin D, schizandrin, schizandrin A, and wuweizisu were identified.

First, we used the UPLC method to analyze components of TJT. As a result, we could identify several components including ephedrine, pseudoephedrine, methylephedrine, methylpseudoephedrine, sinapine, platycodin D, schizandrin, schizandrin A, wuweizisu B (**Figure 1**, **Table 3** and **Supplementary Table S1**).

**Table 3:** Identified components of TJT.

No.	Retention time (min)	Characteristic ion	Herbal constituent	Proposed structure
1	2.951	[M+H]+	*Ephedra sinica Stapf*	(Pseudo) Ephedrine
2	3.094	[M+H]+		Methyle (pseudo) ephedrine
3	3.589	[M+H]+	*Raphanus raphanistrum subsp. sativus (L.) Domin*	Sinapine
4	7.344	[M+H]+	*Platycodon grandiflorus (Jacq.) A.DC.*	Platycodin D
5	13.083	[M+H]+	*Schisandra chinensis (Turcz.) Baill.*	Schisandrol A
6	16.239	[M+H]+		Schizandrin A
7	16.607	[M+H]+		Schizandrin B

### TJT Ameliorates Obesity in HFD/DNFB-Induced Comorbidity Mice

**Figure 2:**
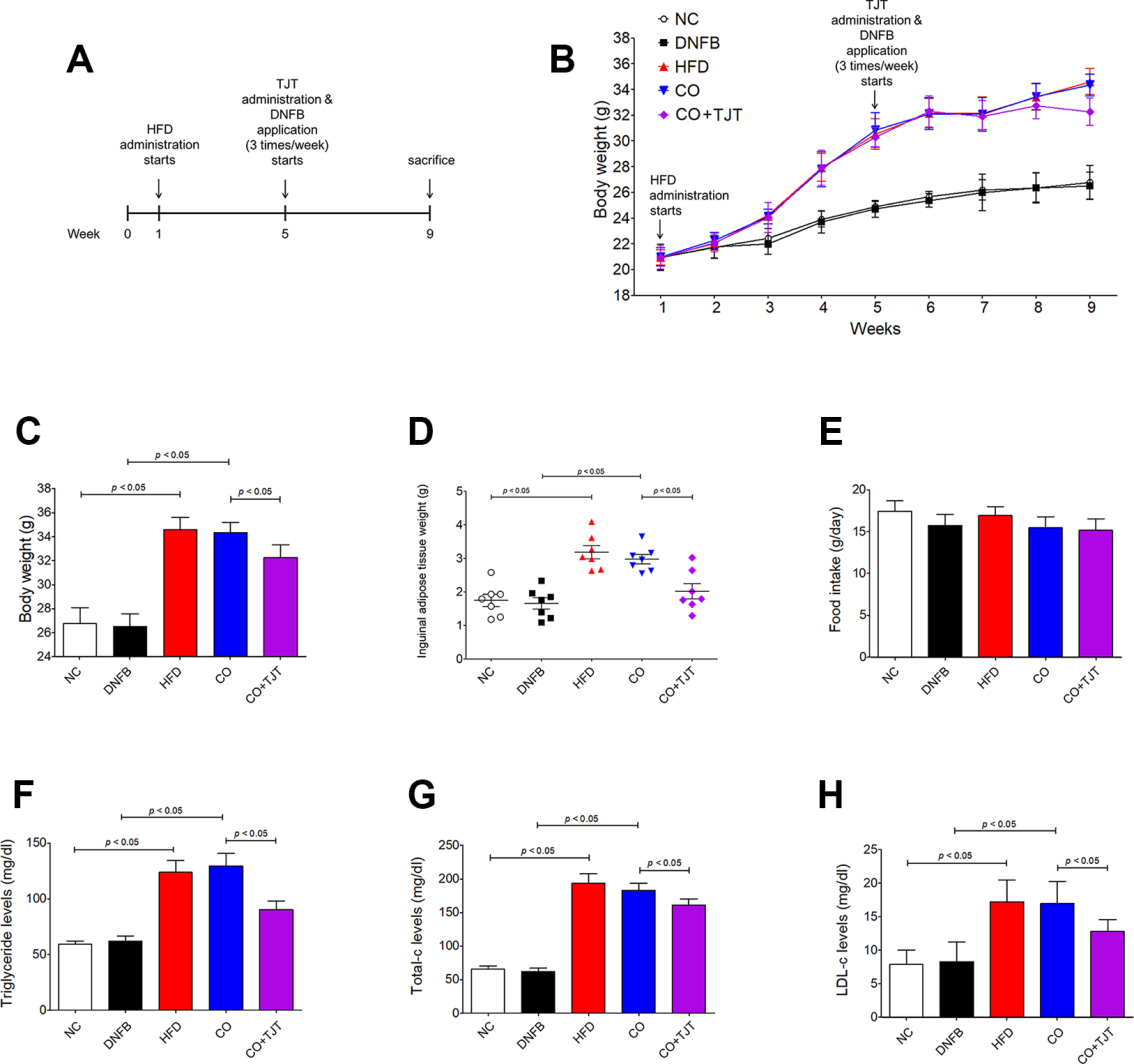
TJT ameliorates obesity-related symptoms in the HFD/DNFB-induced obesity-AD comorbidity mouse model. **(A)** The mice (n = 7 per group) were administered 60% kcal HFD for a total of 8 weeks, while TJT treatment and 0.35% DNFB application (3 times/week) started on week 5. **(B**, **C)** The body weight changes of the mice were measured every week. **(D)** Inguinal adipose tissue weight of mice was compared. **(E)** Food intake was measured. Plasma levels of **(F)** triglyceride, **(G)** total cholesterol, and **(H)** low density lipoprotein cholesterol were measured. The data are represented as the mean ± SEM. NC, normal control group; DNFB, DNFB-induced AD group; HFD, HFD-induced obesity group; CO, HFD/DNFB-induced obesity-AD comorbidity group; CO+TJT, TJT-treated comorbidity group.

Based on previous publications (Kimber et al., 2001; Gould et al., 2003; Katagiri et al., 2007), we established an obesity-AD comorbidity mouse model by administrating 60% kcal HFD and 0.35% DNFB (**Figure 2A**). As seen in **Figure 2B**, 4 weeks of HFD administration resulted in a significant body weight difference between the normal control and HFD-treated mice (24.87 ± 0.43 g vs. 30.52 ± 1.18 g, respectively). At the start of week 5, the HFD-treated mice were randomly divided into 3 groups (n = 7 per group), and 2 groups were fed HFD, 1 group was fed HFD + TJT, and DNFB application was applied three times a week. After 4 weeks of additional HFD/DNFB administration, the body weight difference among the 3 groups were as follows: the NC group, 26.77 ± 1.31 g; CO group, 34.35 ± 0.86 g, and the TJT group, 32.27 ± 1.07 g (**Figures 2B, C**). Furthermore, TJT treatment reduced the weight of inguinal adipose tissues (**Figure 2D**) and several serum parameters such as triglyceride (**Figure 2F**), total cholesterol (**Figure 2G**), and LDL cholesterol (**Figure 2H**), without affecting food intake (**Figure 2E**). On the other hand, to assess whether TJT induced any toxicity in the liver or kidney, we measured serum levels of ALT, AST, and creatinine. As in **Supplementary Figure S1**, TJT did not alter any of these parameters. Thus, we were able to confirm that TJT administration can improve obesity-related symptoms induced by HFD, without displaying neither hepatotoxicity nor nephrotoxicity.

### TJT Improves AD-Related Clinical Symptoms in HFD/DNFB-Induced Comorbidity Mice

**Figure 3:**
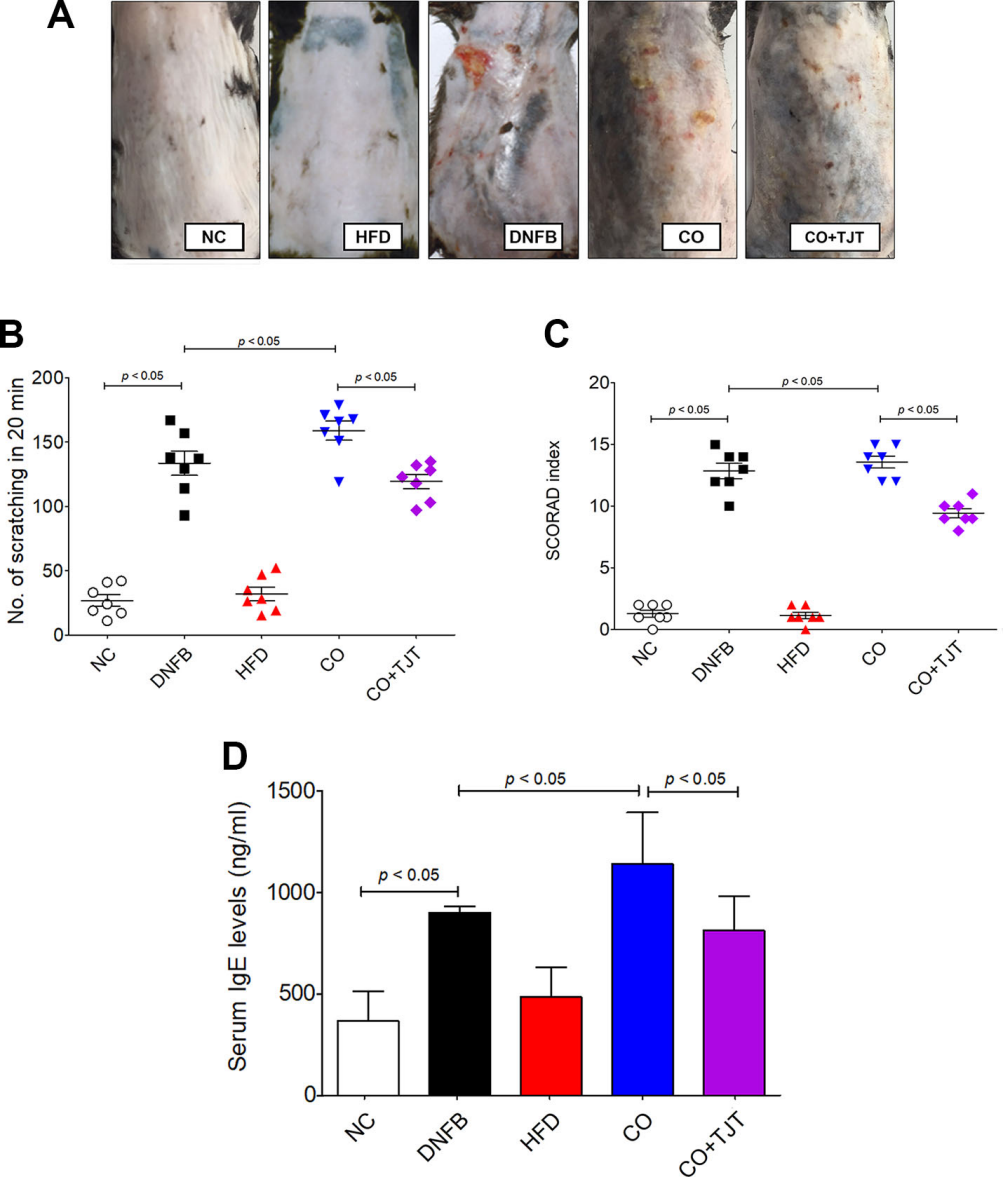
TJT improves AD-related symptoms in the HFD/DNFB-induced obesity-AD comorbidity mouse model. **(A)** The AD-like lesions of the dorsal skin were observed macroscopically, **(B)** the 20-min-scratching behavior and **(C)** the SCORAD index of mice (n = 7 per group) was evaluated. An ELISA assay was performed to evaluate the level of **(D)** serum IgE. The data are represented as the mean ± SEM. NC, normal control group; DNFB, DNFB-induced AD group; HFD, HFD-induced obesity group; CO, HFD/DNFB-induced obesity-AD comorbidity group; CO+TJT, TJT-treated comorbidity group.

When visually observed, HFD/DNFB administration increased AD-like symptoms compared to the DNFB only group (**Figure 3A**). Number of scratching was slightly increased. The DNFB group showed 133.57 ± 9.44 times of scratching in 20 min, While the obesity-AD comorbidity group showed 158.86 ± 7.46 times of scratching during the same time length. The SCORAD scores were also increased significantly (12.77 ± 0.67 Vs. 13.62 ± 0.79) as well (**Figures 3B, C**). However, as shown in **Figure 3A**, TJT remarkably ameliorated the clinical symptoms of the HFD/DNFB-Induced AD. the skin lesions were improved by the TJT treatment (**Figure 3A**), While the number of scratching in 20 min and the SCORAD index was significantly decreased in the TJT group when compared to the CO group (scratching, 158.86 ± 7.46 to 119.43 ± 5.48 times per 20 min; SCORAD index, 8.76 ± 0.98 to 13.62 ± 0.79) (**Figure 3C**).

### TJT Reduces Serum Immunoglobulin E (IgE) in HFD/DNFB-Induced Comorbidity Mice

Because IgE is one of the most important factors in the pathology of AD (Sismanopoulos et al., 2012), the IgE level in total serum was evaluated. As shown in **Figure 3D**, the CO group showed a noticeable increase in the serum IgE level (1,139.22 ± 145.94 ng/mL) when compared with the NC group (367.28 ± 83.14 ng/mL). This IgE level of the CO group was also significantly higher than that of the AD group (900.97 ± 30.26 ng/mL) as well. The difference of IgE level and AD-related clinical symptoms between the DNFB group and CO group showed basic proof to our first hypothesis: obesity aggravates AD. However, the increased IgE was attenuated by TJT (813.79 ± 96.97 ng/mL), showing its potential as a treatment for obesity-AD comorbidity.

### TJT Alleviates Histological Changes in HFD/DNFB-Induced Comorbidity Mice

**Figure 4:**
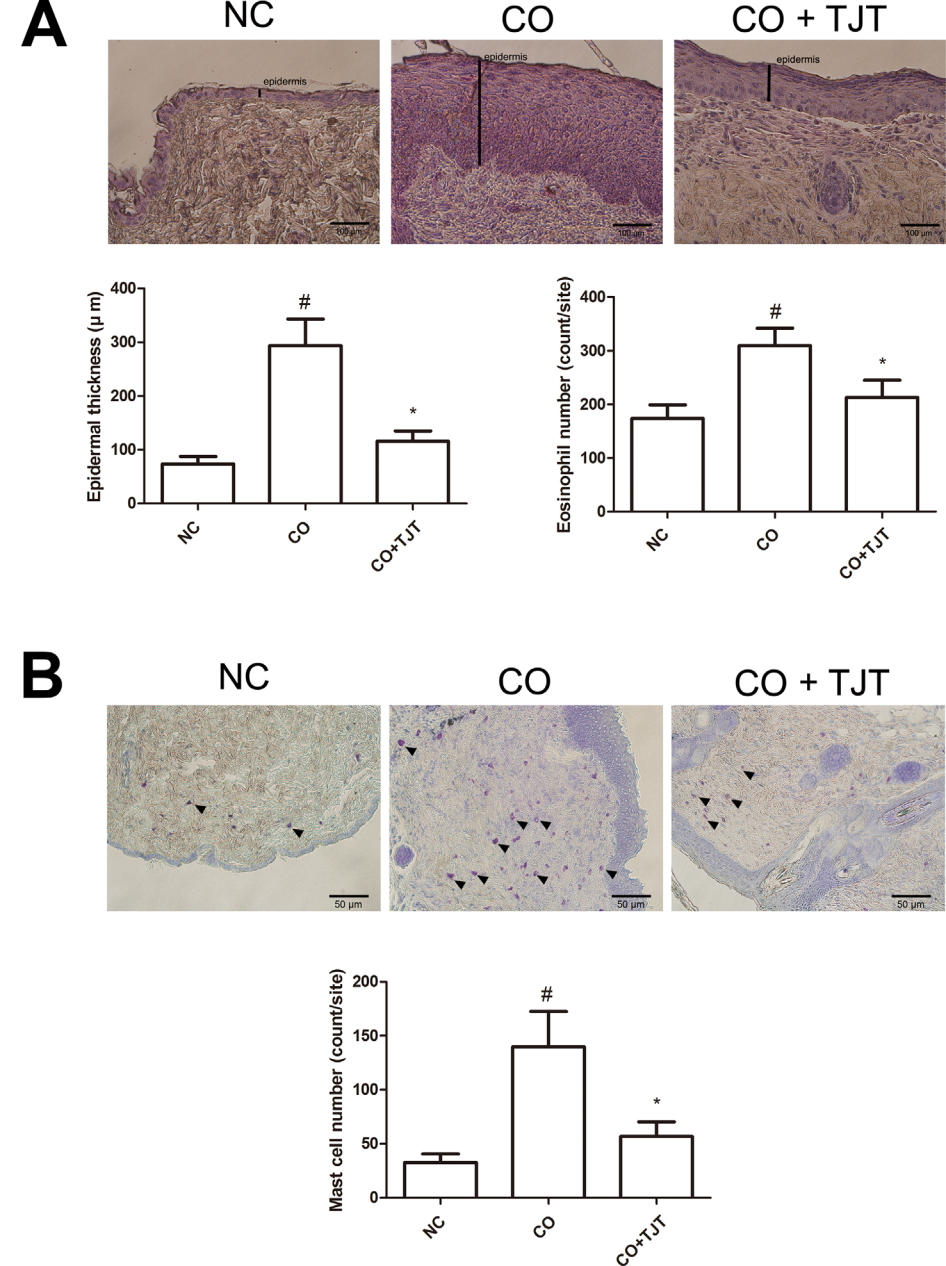
TJT decreases epidermal hyperplasia, eosinophil infiltration, and mast cell infiltration in the skin lesions of the HFD/DNFB-induced obesity-AD comorbidity mouse model. **(A)** An H&E assay was performed and microscopically observed at 400× magnification, and the epidermal thickness and eosinophil number were measured. **(B)** A toluidine blue staining assay was performed and microscopically observed at 200× magnification, and the infiltrated mast cell number was counted. Epidermal thickness, eosinophil number, and mast cell number were evaluated in five random slides per group. The data are represented as the mean ± SEM. ^#^ *P* < 0.05 when compared to NC; *P < 0.05 when compared to CO. NC, normal control group; CO, HFD/DNFB-induced obesity-AD comorbidity group; CO+TJT, TJT-treated comorbidity group.

In order to confirm the protective effect of TJT on obesity-AD comorbidity, we performed an H&E assay. As shown in **Figure 4A** and **Supplementary Figure S2a**, the average epidermal thickness was greater in the CO group than in the NC group (293.94 ± 49.18 μm vs. 73.46 ± 14.15 μm). However, the epidermal hyperplasia was significantly reduced by TJT treatment (115.98 ± 18.70 μm). As mast cell and eosinophil infiltrations are also commonly observed in AD (Stone et al., 1976; Galli and Tsai, 2012; Sismanopoulos et al., 2012; Desai et al., 2013), we next counted the number of eosinophils infiltrated into the skin lesions of mice. The number of infiltrated eosinophils was highly suppressed in the TJT group (213.20 ± 32.04 cells vs. 309.80 ± 32.06 cells in the CO group). Then, we performed a toluidine blue staining assay to evaluate mast cell infiltration in the dermis. Mast cells are closely linked to allergic reactions because they possess a variety of inflammatory mediators such as histamines, which have important roles in AD (Stone et al., 1976; Huber et al., 2002). The increased number of infiltrated mast cells by HFD/DNFB administration was suppressed in the TJT group (**Figure 4B** and **Supplementary Figure S2b**).

### TJT Decreases Pro-Inflammatory Cytokine Levels in HFD/DNFB-Induced Comorbidity Mice

**Figure 5:**
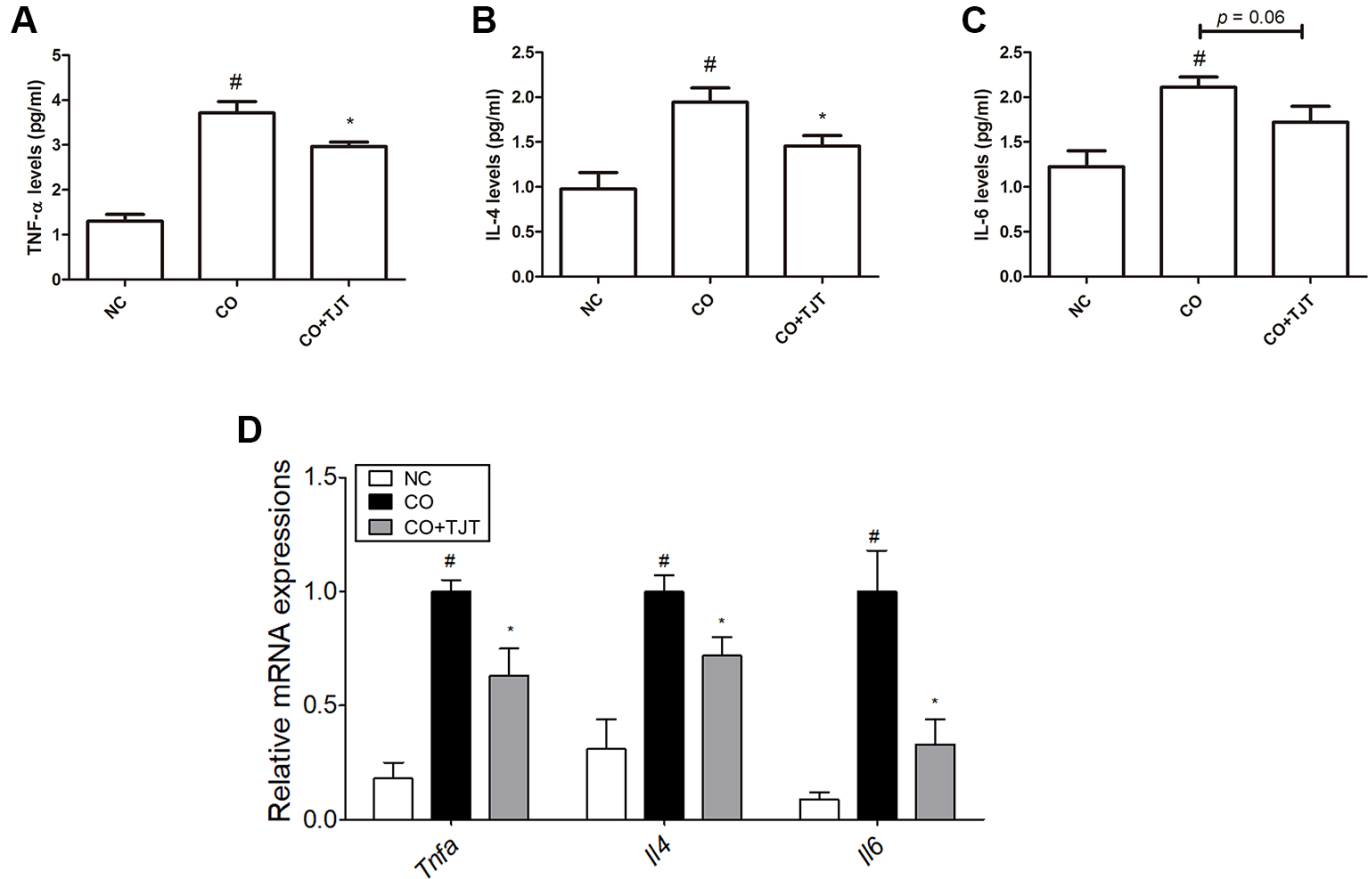
TJT reduces pro-inflammatory cytokines in the HFD/DNFB-induced obesity-AD comorbidity mouse model. An ELISA assay was performed to evaluate the levels of dorsal skin tissue-expressions of **(A)** TNFα, **(B)** IL-4, and **(C)** IL-6. **(D)** A Real-Time RT-PCR assay was performed to evaluate the mRNA levels of *Tnfa*, *Il4* and *Il6* in the dorsal skin tissue of mice (n = 7 per group). The data are represented as the mean ± SEM. ^#^ *P* < 0.05 when compared to NC; *P < 0.05 when compared to CO. NC, normal control group; CO, HFD/DNFB-induced obesity-AD comorbidity group; CO+TJT, TJT-treated comorbidity group.

Besides elevated IgE several inflammatory mediators such as nuclear factor-κB (NF- κB) (Huber et al., 2002), interleukins (IL)-4 (Furue et al., 1989) and 6 (Toshitani et al., 1993), and mitogen-activated protein kinases (MAPKs) (Pastore et al., 2005) are also known to participate in the pathology of AD. Therefore, we did further experiments on cytokine expressions in the dorsal skin. The levels of IL-4 and 6 and tumor necrosis factor-alpha (TNFα) were evaluated with an ELISA assay. As seen in **Figures 5A–C**, IL-4 and IL-6 and TNFα were highly up-regulated by HFD/DNFB administration (1.94 ± 0.16 pg/mL, 2.11 ± 0.12 pg/mL, 3.71 ± 0.25 pg/mL, respectively) compared to those in the NC group (0.98 ± 0.18 pg/mL, 1.23 ± 0.18 pg/mL, 1.30 ± 0.15 pg/mL, respectively), while TJT treatment successfully suppressed the levels of IL-4 and TNFα (1.46 ± 0.12 pg/mL, 2.97 ± 0.10 pg/mL, respectively) although it could not decrease the level of IL-6 (*P* = 0.06, 1.72 ± 0.18 pg/mL). In addition, the mRNA levels of these cytokines were decreased by TJT treatment (Tnfa, 0.63-fold; Il4, 0.72-fold; Il6, 0.33-fold) (**Figure 5D**).

### TJT Down-Regulates Protein Expressions of NF-κB and MAPKS in HFD/DNFB-Induced Comorbidity Mice

**Figure 6:**
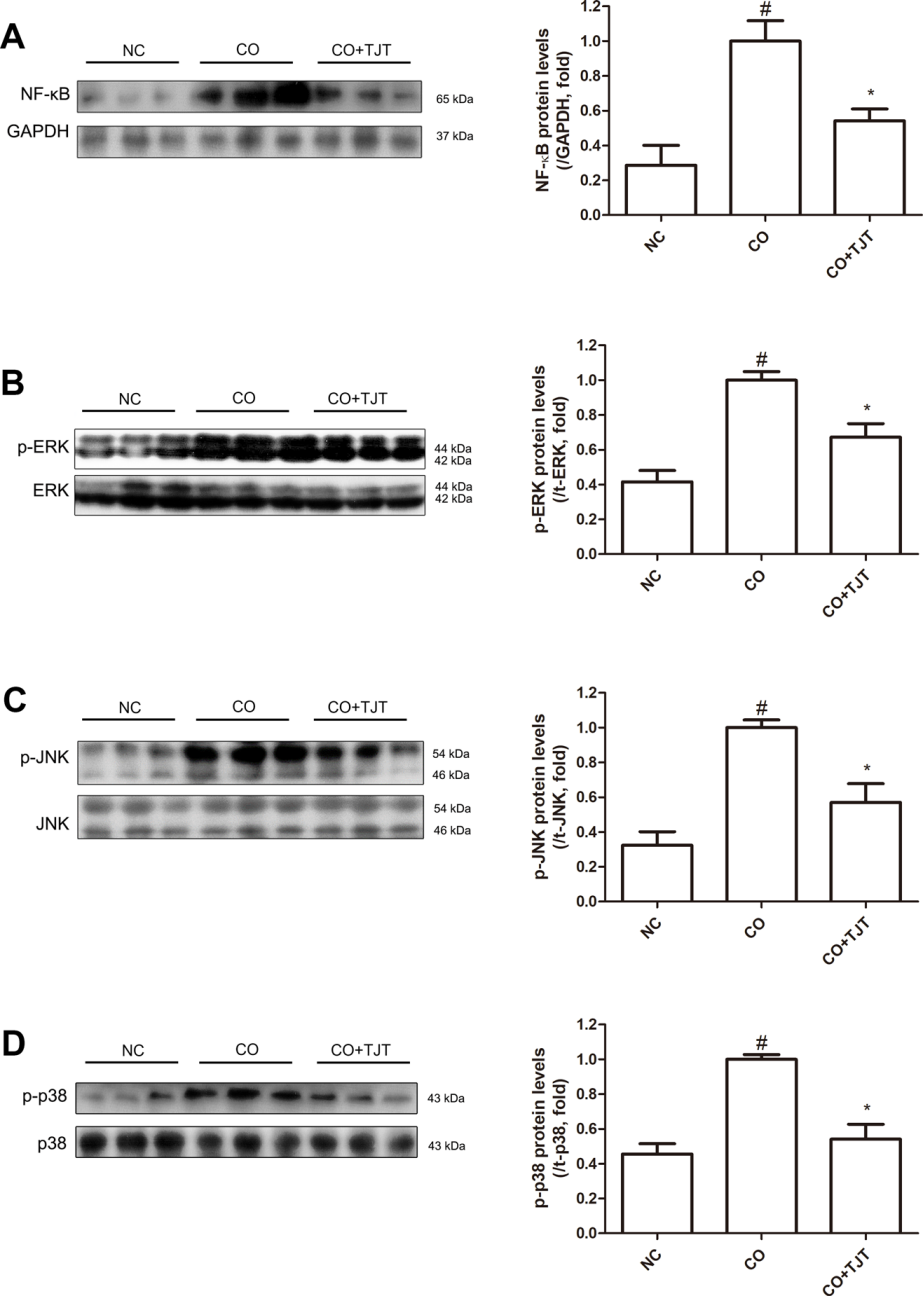
TJT suppresses activation of nuclear NF-κB and MAPKs in the HFD/DNFB-induced obesity-AD comorbidity mouse model. Western blot analyses were done to evaluate the levels of **(A)** nuclear NF-κB **(A)**, **(B)** p-ERK, **(C)** p-JNK, **(D)**, and p-p38. The data are represented as the mean ± SEM of three or more experiments. ^#^ *P* < 0.05 when compared to NC; *P < 0.05 when compared to CO. NC, normal control group; CO, HFD/DNFB-induced obesity-AD comorbidity group; CO+TJT, TJT-treated comorbidity group.

The CO group showed 3.50-fold higher expressions of nuclear NF-κB than that of the NC group. However, in the TJT group, this key inflammatory factor was suppressed down to 54.2% of that of CO mice (**Figure 6A**). In addition, phosphorylation of the MAPKs was also reduced by TJT (**Figure 6B–D**). While ERK, JNK, and p38 MAPKs were highly activated in the CO group when compared to the NC group, however, in the TJT group, phosphorylation levels of these three MAPKs were reduced (p-ERK/ERK, 0.67-fold; p-JNK/JNK, 0.57-fold; p-p38/p38, 0.54-fold), implying suppressed inflammation by TJT treatment.

### TJT Decreases HIF-1α Expression in HFD/DNFB-Induced Comorbidity Mice

**Figure 7:**
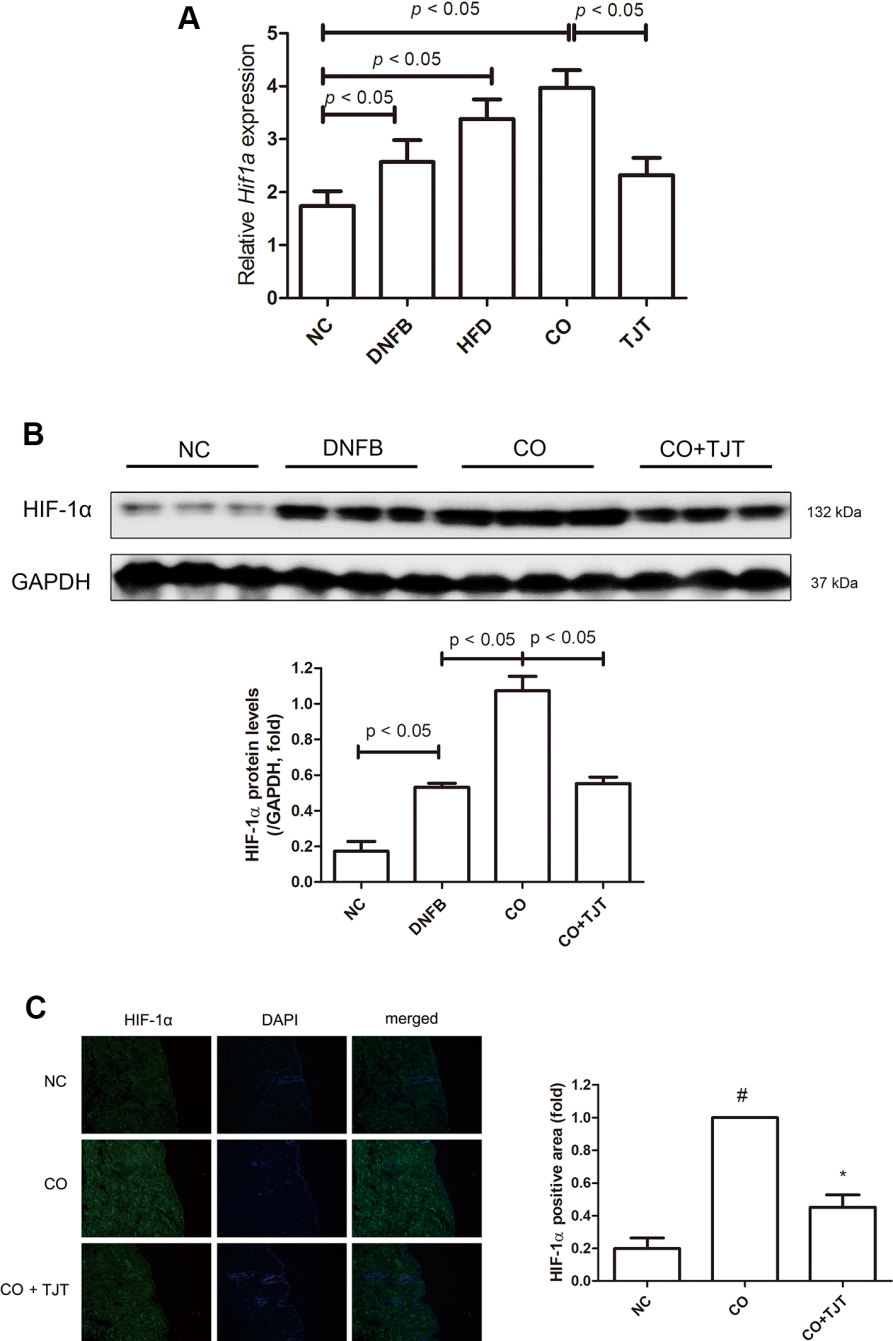
TJT decreases HIF-1α expression in the HFD/DNFB-induced obesity-AD comorbidity mouse model. **(A)** A western blot assay and **(B)** immunofluorescence assay were done to examine HIF-1α expressions (IF magnification ×400). HIF-1α positive area was evaluated in five random slides per group using Image J. The data are represented as the mean ± SEM of three or more experiments. ^#^ *P* < 0.05 when compared to NC; *P < 0.05 when compared to CO. NC, normal control group; DNFB, DNFB-induced AD group; CO, HFD/DNFB-induced obesity-AD comorbidity group; CO+TJT, TJT-treated comorbidity group.

HIF-1α, a transcription complex that has a key role in hypoxic conditions, can also be expressed in normoxic inflammation (Zhou et al., 2003). Several studies report this factor has a crucial role in inflammatory skin diseases as well (Simonetti et al., 2006; Viemann et al., 2007; Kim et al., 2011a). We therefore evaluated the HIF-1α expression in the dorsal skin lesions. As shown in **Figure 7A**, H*if1a*, the gene which transcripts HIF-1α, was markedly increased in the CO group. This elevation of *Hif1a* was suppressed by TJT treatment. Similar results were observed in western blot assays. The protein expression of HIF-1α was significantly altered in the skin tissues of CO group than in those of DNFB group (2.02-fold increase), which already showed elevated HIF-1α levels when compared to the NC group (**Figure 7B**). Therefore, we assumed HIF-1α might be the crucial factor which worsens the AD-like condition in obesity-AD comorbidity. While protein expression of HIF-1α was up-regulated by HFD/DNFB administration as expected, this increase was suppressed in the TJT group shown by western blot assay. Next, we performed an IF assay to examine changes in HIF-1α in the dorsal skin tissue. As seen in **Figure 7C**, the IF assay showed that HIF-1α expression, mostly located in the cytosol close to the nucleus, was highly increased in the CO group. The elevated HIF-1α was reduced by TJT administration.

### TJT Ameliorates Obesity-AD Comorbidity by Regulating HIF-1α

**Figure 8:**
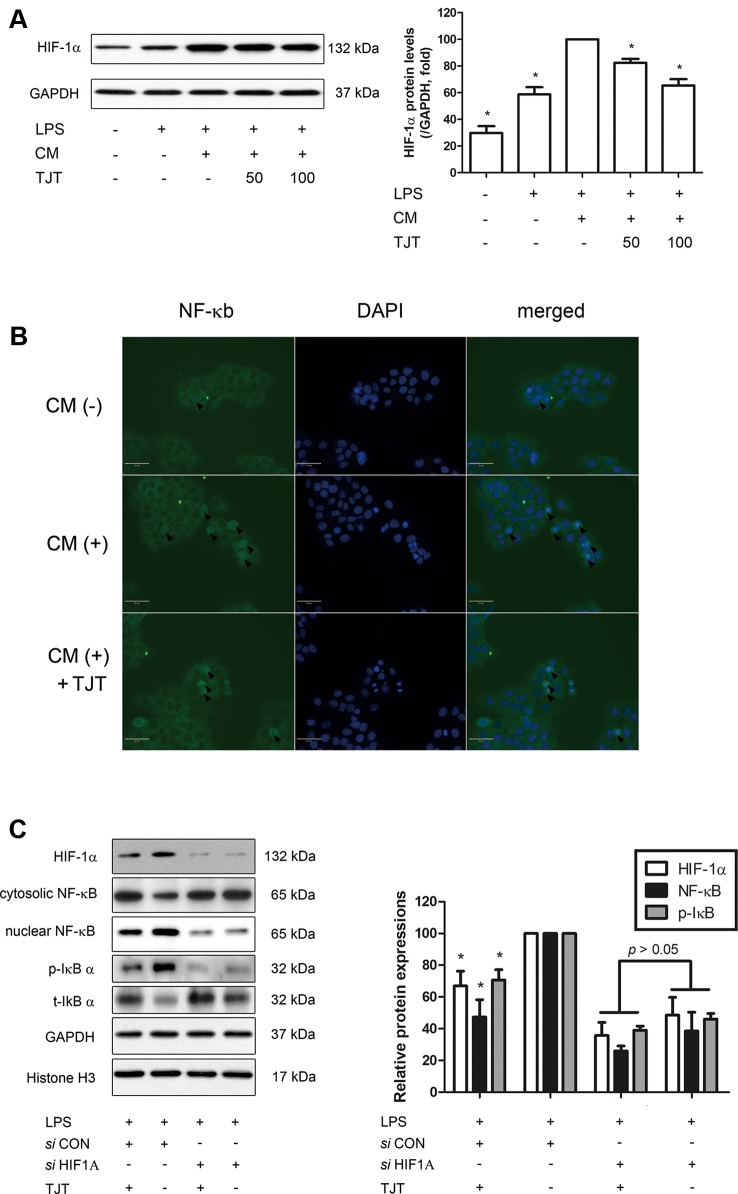
TJT improves obesity-AD comorbidity *via* HIF-1α regulation. **(A)** A western blot assay was performed to examine HIF-1α expression in an obesity-AD comorbidity vitro model of LPS-activated, adipocyte CM-treated HaCaT cells. **(B)** Localization of NF-κB is shown by IF staining of adipocyte CM-treated HaCaT cells. **(C)** Expression levels of HIF-1α, cytosolic and nuclear NF-κB and p-IκB were determined by western blot assays. The data are represented as the mean ± SEM of three or more experiments. HIF-1α was normalized to GAPDH, p-IκB was normalized to t-IκB, and nuclear NF-κB was normalized to histone H3. *P < 0.05 when compared to LPS and CM-treated HaCaT cells in **(A)**; *P < 0.05 when compared to LPS and control siRNA-treated HaCaT cells in **(B)**.

To elucidate the role of HIF-1α in the pathogenesis of obesity-AD comorbidity, we established a comorbidity *in vitro* model. To mimic the comorbid condition of obesity and AD, HaCaT human keratinocytes were cultured in adipocyte conditioned media (CM) and inflammatory response was induced by treating lipopolysaccharide (LPS). As shown in **Figure 8A**, LPS-induced elevation of HIF-1α expression was further increased when the cells were cultured in CM, implying the impact of adipocytes on the inflammatory response of keratinocytes. An IF staining assay revealed that CM treatment induced the localization of NF-κB into the nucleus of HaCaT cells, which was inhibited in TJT-treated cells (**Figure 8B**). TJT treatment regulated NF-κB-mediated inflammatory signaling by suppressing HIF-1α in the vitro model of obesity-AD comorbidity in a dose-dependent manner.

Next, we used the small interfering RNA (siRNA) method to confirm the role of HIF-1α during the action mechanism of TJT in ameliorating obesity-AD comorbidity. When *HIF1A* gene was knocked down by *siHIF1A*, TJT failed to decrease inflammatory markers such as nuclear NF-κB and p-IκB (**Figure 8B**). These results suggest the improving of TJT on obesity-AD comorbidity is dependent, at least partially, on HIF-1α pathway.

## Discussion

Obesity and AD are two separate diseases both etiologically and pathologically. While obesity is a metabolic disorder with excess accumulation of energy, AD is a chronic inflammatory illness of the skin. However, these two diseases often occur together. In addition to the prevalence, the severeness of the problem is quite affected by each other (Visness et al., 2009; Silverberg and Simpson, 2014). However, the exact correlation mechanism of the two is yet to be discovered. Katagiri et al. tried to provide evidence for the effect of obesity on inflammatory responses by the administration of trinitrochlorobenzene (TNCB) or ovalbumin in diet induced obese mice (2007). Based on this experiment model, we established four different obesity-AD comorbidity mouse models. HFD-fed and 0.15% DNFB-applied BALB/c mice did not show any weight gain. On the other hand, obesity was certainly induced in C57BL/6J mice, but 0.15% DNFB administration failed to show AD-like dermatitis. By application of 0.5% DNFB, unexpected weight loss in the mice was occurred. Finally, with C57BL/6J mice and administration of HFD plus 0.35% of DNFB, we successfully induced both obesity and AD. The results showed that HFD/DNFB application induced body weight gain and AD-like skin lesions. In addition, serum parameters and clinical AD symptoms was increased in the comorbidity model.

TJT is an herbal remedy which originally is used to treat ‘exterior-cold disease by a cold in the esophagus of Taeum type patients (Lee et al., 2009). However, several experimental and clinical reports support the use of TJT as treatment for both obesity (Park and Cho, 2004; Kim et al., 2011b; Park, S., et al., 2013) and AD (Sun et al., 2004). Therefore, we expected TJT to be a potential treatment for obesity-AD comorbidity management. As in our hypothesis, TJT treatment for 4 weeks showed remarkable improvement on HFD/DNFB-induced obesity and AD-like skin lesions. The positive effects of TJT also influenced other changes caused by HFD and DNFB. The epidermal thickness was reduced; eosinophil/mast cell infiltration was decreased, and IgE, TNF-α, IL-4, IL-6, NF-κB, and MAPK phosphorylation were all suppressed. Furthermore, HIF-1α was highly down-regulated by TJT treatment.

Identified first in the 1960s (Johansson and Bennich, 1967), IgE was recognized to be a key factor in several allergic diseases due to its high level in such patients (Johansson, 1969). AD also is not an exception because allergens are taken up by dendritic cells, followed by binding of the allergen-specific IgE to the receptors (FcεRI) in mast cells, which are then stimulated to degranulate and release pro-inflammatory molecules (Cookson, 2004). This important regulator in AD is surprisingly increased in obesity as well, a disease that seemed to be non-relevant to inflammation or allergy (Visness et al., 2009). This fact leaves us a hint for the correlation between obesity and AD. Supporting this clinical phenomenon, some studies have shown the relationship of IgE and obesity in *in vivo* and *in vitro* experiments (Nagai et al., 2012; Ramalho et al., 2012). In the same context, our newly established obesity-AD comorbidity model showed elevated IgE levels by HFD/DNFB application. TJT treatment was able to ameliorate the changed IgE levels and also the other related factors such as TNF-α, IL-4, 6, and NF-κB. IL-4, a pro-inflammatory cytokine, is secreted from the T helper 2 cells as an inflammatory response resulting in IgE production, which leads to degranulation of mast cells (Friedmann, 2006). IL-6 is a cytokine released by various immune-related cells, including macrophages, T cells, and B cells (Brandt and Sivaprasad, 2011), while TNF-α is a cytokine which is produced mostly by macrophages and involved closely in inflammation, especially in the acute phase reaction (Gahring et al., 1996).

NF-κB and MAPKs were also up-regulated in the obesity-AD comorbid mice. NF-κB, a transcription factor which controls cytokine production and cell survival, is one of the key factors in the immune response process (Tak and Firestein, 2001; Lawrence, 2009). MAPKs are involved in the pathology of AD as they are regulated by IgE and can induce histamine release or HIF-1α accumulation (Sumbayev et al., 2010). In this study, NF-κB and MAPKs were highly marked in the HFD/DNFB-treated group, suggesting severe comorbidity of obesity and AD. In the TJT group, the activation of these factors was decreased close to the levels in the NC group.

HIF-1 was originally recognized as an oxygen homeostasis factor with the known purpose to regulate oxygen delivery in mammals (Semenza, 1999). Among its biological functions, recent perspectives suggest the role of HIF-1α in inflammation to be important. As IgE activates the IgE receptor FcεRI in basophils, two main members of the MAPK chain, ERK and p38, start to induce the protein accumulation of HIF-1α (Sumbayev et al., 2009). Generally, the role of HIF-1α in AD is not widely known; however, considering its response to IgE and roles in inflammation, HIF-1α could be a participant. Other studies also imply the possibility that it is a key regulator in AD. HIF-1α is known a key regulator in immune responses (Rezvani et al., 2007; Jang e t al., 2013; Leire et al., 2013). Such previous reports cannot reveal the exact role of HIF-1α in AD; however, they briefly suggest the possibility of its correlation. Our study showed remarkably high expression of HIF-1α in the HFD/DNFB-induced obesity-AD comorbidity mice model, supporting its involvement in this certain status. And by TJT treatment, HIF-1α was attenuated to a similar level as in the normal mice. These results indicate the role of HIF-1α in obesity-AD comorbidity, and TJT can successfully improve the comorbidity disease by regulating HIF-1α.

**Figure 9:**
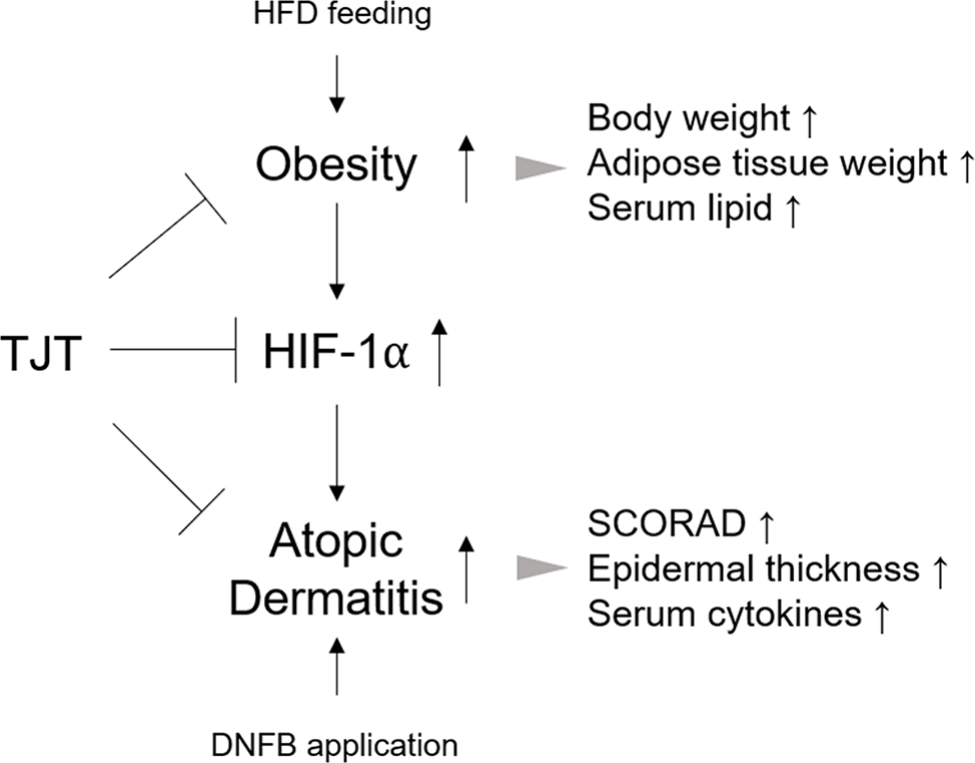
TJT alleviates obesity-AD comorbidity via regulation of HIF-1a.

Nevertheless, our study only provides a hint; the actual role of HIF-1α in obesity-AD comorbidity remains unclear. Some suggestive studies may support the idea that HIF-1α links obesity and AD. Several reports introduce the connection between HIF-1α and cytokines such as IL-4, IL-5, IL-13 (Crotty Alexander et al., 2013; Mo et al., 2014), and other studies show that these cytokines are elevated in obese individuals (El-Wakkad et al., 2013; Schmidt et al., 2015). Furthermore, a study by Desai et al. reports that IL-5 is increased in obese individuals with severe asthma (2013). Regarding the pathological similarity between asthma and AD, the role of HIF-1α and cytokines, especially IL-5 in particular, may give an answer to the unrevealed mechanism of obesity-AD comorbidity. Considering the role of HIF-1α in necrosis suggest another possibility. In necrotic conditions of skin tissue, impaired O_2_ supply promote HIF-1α stabilization (Lee et al., 2007). Infiltrated immune cells, such as mast cells or eosinophils may also contribute to HIF-1α in skin lesions (Walczak-Drzewiecka et al., 2008; Mo et al., 2014). In these cases, the reduction of HIF-1α expressions by TJT could be a secondary effect resulting from alleviation of inflammation. Our results from an *in vitro* model of obesity-AD comorbidity-mimicking conditions suggested the possible role of HIF-1α in the action mechanism of TJT is crucial (**Figure 9**), but still further intense studies, perhaps using HIF-1α knock-out mice, must be proceeded in order to investigate the underlying mechanism of TJT on HIF-1α.

## Conclusion

Obesity and AD are separate diseases with different clinical symptoms, but closely linked epidemiologically. Despite the prevalence of obesity-AD comorbidity, its exact pathological mechanism remains to be elucidated. In our study, we propose a basic *in vivo* model of obesity-AD comorbidity through HFD/DNFB administration, and discovered HIF-1α as one of the possible links between the two diseases. Although we showed TJT could successfully improve the symptoms of obesity-AD comorbidity by regulating HIF-1α, the specific role of HIF-1α in the pathology of obesity-AD and the detailed regulatory mechanism of TJT require further investigation. Taken all together, TJT might be useful in obesity-AD comorbidity treatment because it improves clinical symptoms, and this may be due to its HIF-1α regulating effect.

## Data Availability Statement

The raw data supporting the conclusions of this article will be made available by the authors, without undue reservation, to any qualified researcher.

## Ethics Statement

All experimental protocols involving the use of animals conform to the NIH guidelines (Guide for the Care and Use of Laboratory Animals, 8th edition). Animal Care and Use Committee of the Institutional Review Board of Kyung Hee University (confirmation number: KHUASP(SE)-12-036) has approved all the animal experiments.

## Author Contributions

JP and J-YU designed the protocol and prepared the manuscript. JP, D-HY, and JK performed the experiments. JP, D-HY, and JK curated data. HJK and KSA provided technical and material support. J-YU was in charge of conducting the whole experiment and proofreading the manuscript. All authors approved the final version to be published.

## Funding

This work was supported by the National Research Foundation of Korea (NRF) grant funded by the Korea government (MSIP) (NRF-2015R1A4A1042399, 2018R1A2A3075684 and 2018R1D1A1B07049882).

## Conflict of Interest

The authors declare that the research was conducted in the absence of any commercial or financial relationships that could be construed as a potential conflict of interest.
