# Supplementary material for: Taeumjowi-tang, a Traditional Korean Sasang Remedy, Improves Obesity-Atopic Dermatitis Comorbidity by Regulating Hypoxia-Inducible Factor 1 Alpha
Source: Front Pharmacol. 2019 Dec 20;10:1458. doi: 10.3389/fphar.2019.01458 (PMC6933016; doi:10.3389/fphar.2019.01458)
Supplement: Supplementary file 1 [file DataSheet_1.pdf]

Supplementary Materials

1 Supplementary Figure S1

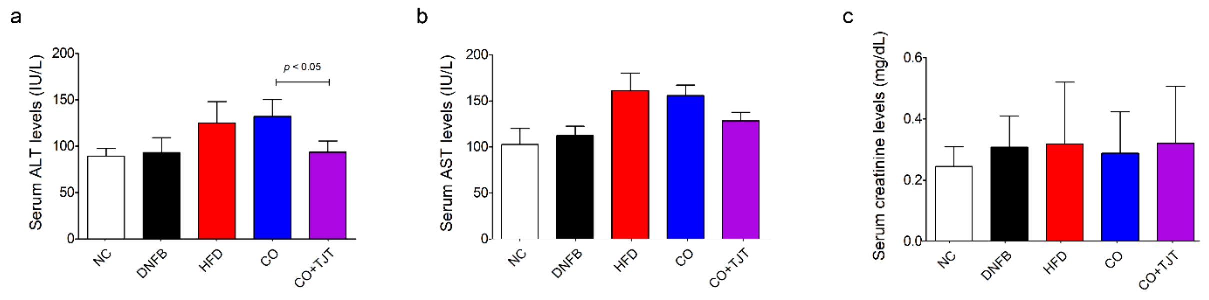

Supplementary Figure S1. : Effect of TJT on hepatotoxicity and nephrotoxicity in the HFD/DNFB-induced obesity-AD comorbidity mouse model. Serum levels of (a) ALT, (b) AST, and (c) creatinine was measured. *$P < 0.05$ when compared to CO. NC, normal control group; DNFB, DNFB-induced AD group; CO, HFD/DNFB-induced obesity-AD comorbidity group; CO+TJT, TJT-treated comorbidity group.

2 Supplementary Figure S2

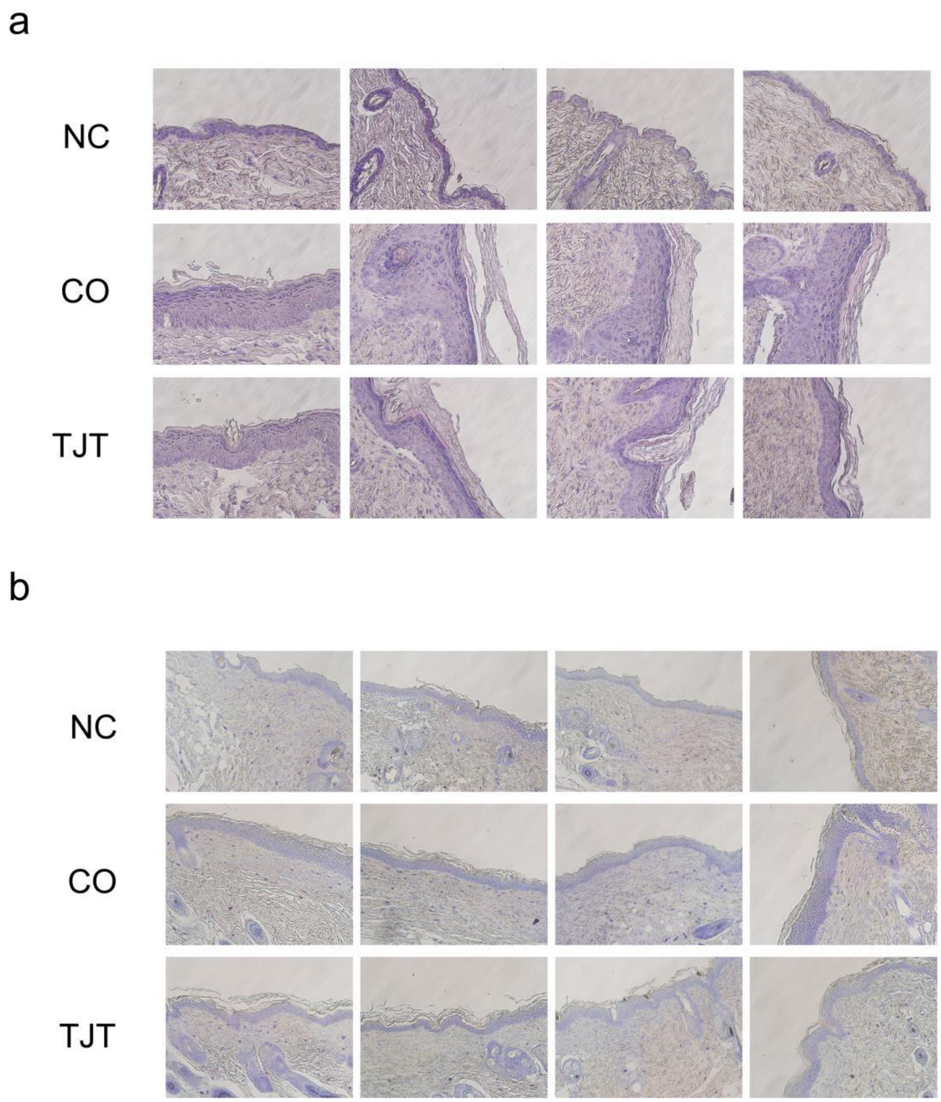

Supplementary Figure S2. : Effect of TJT on histological changes in skin lesions of the HFD/DNFB-induced obesity-AD comorbidity mouse model. (a) An H&E assay was performed and microscopically observed at 400× magnification. (b) A toluidine blue staining assay was performed and microscopically observed at 200× magnification. NC, normal control group; CO, HFD/DNFB-induced obesity-AD comorbidity group; TJT, TJT-treated comorbidity group.

Supplementary Table S1. UPLC conditions

<table>
<tr><th colspan="2">Parameter</th><th>Condition</th></tr>
<tr><td rowspan="6">UPLC</td><td>Instrument</td><td>Thermo Scientific Vanquish UHPLC system</td></tr>
<tr><td>Column</td><td>ZORBAX Eclipse Plus C18 (2.1 x 100mm, 1.8 µm)</td></tr>
<tr><td>Column temperature</td><td>40 ℃</td></tr>
<tr><td>Mobile phase</td><td>(A) 0.1% formic acid in Water
(B) 0.1% formic acid in ACN</td></tr>
<tr><td>Gradient conditions</td><td><table>
<tr><td>Time (min)</td><td>Flow</td><td>B (%)</td></tr>
<tr><td>0</td><td>0.5</td><td>5</td></tr>
<tr><td>0.8</td><td>0.5</td><td>5</td></tr>
<tr><td>2.5</td><td>0.5</td><td>20</td></tr>
<tr><td>5.5</td><td>0.5</td><td>32</td></tr>
<tr><td>8</td><td>0.5</td><td>38</td></tr>
<tr><td>10</td><td>0.5</td><td>45</td></tr>
<tr><td>12</td><td>0.5</td><td>60</td></tr>
<tr><td>16</td><td>0.5</td><td>95</td></tr>
<tr><td>20</td><td>0.5</td><td>100</td></tr>
<tr><td>23</td><td>0.5</td><td>100</td></tr>
<tr><td>23.5</td><td>0.5</td><td>5</td></tr>
<tr><td>27</td><td>0.5</td><td>5</td></tr>
</table></td></tr>
<tr><td>Injection volume</td><td>2 µl</td></tr>
<tr><td rowspan="4">ESI-MS</td><td>Instrument</td><td>Triple ToF MS</td></tr>
<tr><td>Ion spray voltage floating</td><td>5500 V(positive mode) / -4500 V (negative mode)</td></tr>
<tr><td>Temperature</td><td>550 ℃</td></tr>
<tr><td>Mass scan range</td><td>80 ~1500 m/z</td></tr>
</table>
